# Impressive Performance: New Disposable Digital Ureteroscope Allows for Extreme Lower Pole Access and Use of 365 μm Holmium Laser Fiber

**DOI:** 10.1089/cren.2016.0051

**Published:** 2016-06-01

**Authors:** Raymond J. Leveillee, Emily Fell Kelly

**Affiliations:** Department of Urology, Florida Atlantic University, Charles E. Schmidt College of Medicine, Bethesda Hospital East, Boynton Beach, Florida.

## Abstract

***Background:*** Since the development of the first flexible ureteroscope, in 1964, technological advances in image quality, flexibility, and deflection have led to the development of the first single-use digital flexible ureteroscope, LithoVue™ (Boston Scientific, Marlborough, MA). With respect to reusable fiber-optic and now digital ureteroscopes, there is an initial capital cost of several thousand dollars (USD) as well as, controversy regarding durability, the cost of repairs and the burdensome reprocessing steps of ureteroscopy. The single-use LithoVue eliminates the need for costly repairs, the occurrence of unpredictable performance, and procedural delays. Renal stones located in the lower pole of the kidney can be extremely challenging as extreme deflections of greater than 160° are difficult to maintain and are often further compromised when using stone treatment tools, such as laser fibers and baskets. This case describes an initial use of the LithoVue digital disposable ureteroscope in the effective treatment of lower pole calculi using a 365 μm holmium laser fiber.

***Case Report:*** A 35-year-old female, with a medical history significant for chronic bacteriuria, and recurrent symptomatic culture proven urinary tract infections, underwent localization studies. Retrograde ureteropyelography demonstrated two calcifications adjoining, measuring a total of 1.4 cm, overlying the left renal shadow. Urine aspirated yielded clinically significant, >100,000, *Escherichia coli* and *Streptococcus anginosus* bacteriuria, which was felt to be originating from the left lower calix. This case used the newly FDA-approved LithoVue flexible disposable ureteroscope. The two stones were seen using the ureteroscope passed through an ureteral access sheath in the lower pole calix. A 365 μm holmium laser fiber was inserted into the ureteroscope and advanced toward the stones. There was no loss of deflection as the ureteroscope performed reproducibly. The laser was used for more than 4000 pulses at 15 W, producing mucoid debris and fragments. A 1.9F nitinol basket was, then, used to extract the fragments, and the patient was rendered stone free. Treatment success was confirmed by plain abdominal film obtained 1 week after stent removal.

***Conclusion:*** The LithoVue system single-use digital flexible ureteroscope provides an economical advantage over both reusable digital and fiber-optic ureteroscopes. The LithoVue system uses the enhanced image resolution of the digital complementary metal oxide semiconductor imager, similar to other reusable digital ureteroscopes, while maintaining the small ureteroscope size of a flexible fiber-optic ureteroscope, allowing for consistent and effective lower pole access. Deflection characteristics are maintained even when thicker laser fibers are passed through the working channel.

## Background

The controversy behind the cost of repairs and the burdensome reprocessing steps of ureteroscopy has kept the procedure from becoming the standard of care for the treatment of kidney stones. Durability has been a major downfall of the reusable ureteroscopes on the market. The number one risk factor for ureteroscope failure is the number of uses and shortened case life by previous repair.^1^ A study performed by Knudsen et al. showed that the average number of cases before repair for each fiber-optic ureteroscope ranged from 5.3 (DUR-8E) to 18 (URF-P5).^2^ Ketul et al. demonstrated the durability of digital ureteroscopes where the DUR-D performed 11.25 cases and the Olympus URF-V performed 14 cases on average between repairs.^3^ El-Husseiny et al. reported that the DUR-D overtime had a decreased light intensity and the deflection capacity, which deteriorated with each additional use.^4^ Carey et al. showed the costs of maintaining refurbished ureteroscopes. On average each refurbished ureteroscope lasts 6.9 cases. Resulting in the cost of $129,635.65 in refurbishment costs, for 220 cases, which does not include the initial cost of the ureteroscope, the administrative cost of cleaning, storing, and handling the ureteroscope, and the operative costs endured when a ureteroscope is not available secondary to damage.^1,5^ The single-use LithoVue™ eliminates the need for these costly repairs, the occurrence of unpredictable performance, and procedural delays. The cost of the system is $1000 per ureteroscope/case.^6^ Using the digital CMOS, complementary metal oxide semiconductor, platform, high-resolution images are produced, providing an advantage over the conventional, nondigital, fiber-optic ureteroscopes, which produce granular images.^3^

## Case Report

A 35-year-old female, with a medical history significant for chronic stone disease, chronic bacteriuria, recurrent urinary tract infections (UTIs), and a previous percutaneous nephrostolithotomy, presented to the office complaining of UTI symptoms once stopping antibiotic treatment. The decision to proceed with localization studies was made to determine whether the presence of known stones in the lower pole of the left kidney was the nidus of infection. A cystourethroscopy, bilateral renal wash and bladder wash for cultures, and a bilateral retrograde ureteropyelogram were performed to define anatomy. Retrograde pyelography demonstrated two calcifications adjoining to each other, measuring a total of 1.4 cm, overlying the left renal shadow. There appeared a greater than 180° turn from the ureter and narrowing of the infundibulum. Urine aspirated from the left kidney yielded clinically significant, >100,000, *Escherichia coli* and *Streptococcus anginosus*, which was clinically felt to be from the left lower pole calculi. The sample from the right kidney was sterile. The patient was empirically treated with 240 mg of gentamicin and 1 g of cefepime in the inpatient setting and was discharged home on a 14-day course of nitrofurantoin. Urine culture performed at 3 weeks follow-up yielded continued bacteriuria, thus the decision to undergo diagnostic left ureterorenoscopy and a laser lithotripsy with stone extraction was made.

**FIG. 1.**
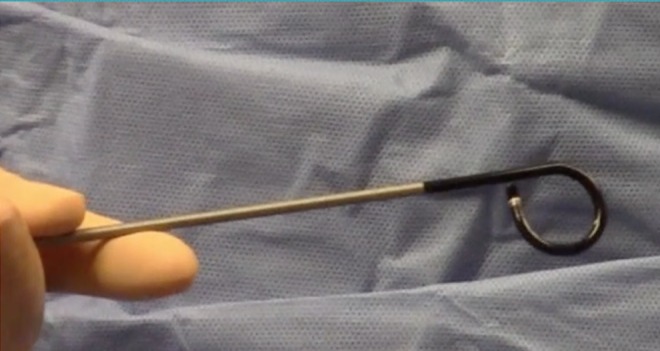
LithoVue out of the package reliable 270° deflectability at the start of each case.

**FIG. 2.**
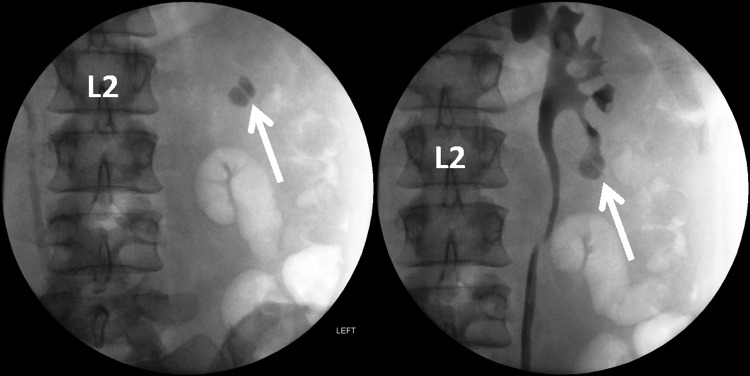
Scout photo, on *left*, and retrograde ureteropyelogram, on *right*, revealing renal calculi in lower pole of left kidney (*white arrow*) located at the infundibular pelvic angle requiring roughly a 180° ureteroscope approach.

**FIG. 3.**
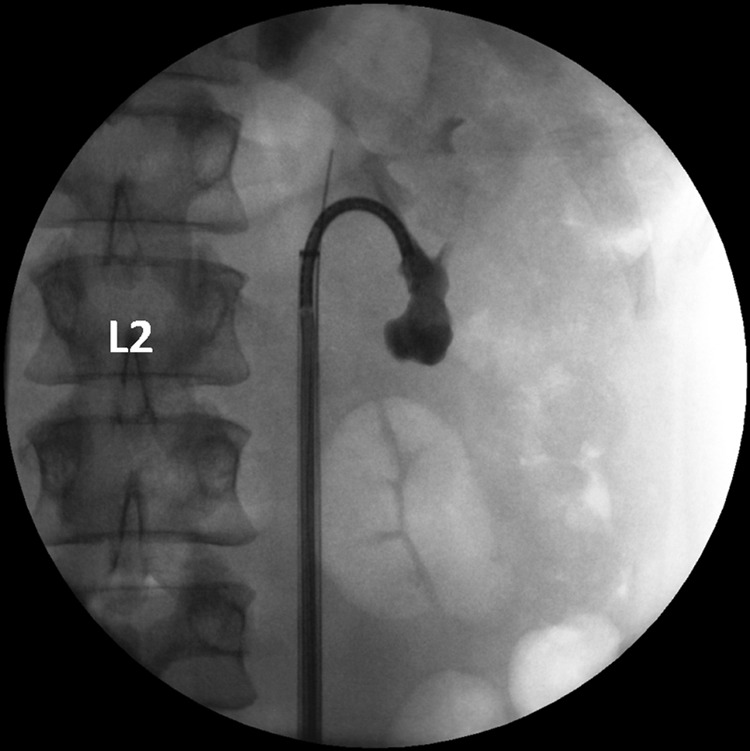
LithoVue with deflection into the lower pole of the left kidney.

This case used the newly FDA-approved LithoVue flexible disposable ureteroscope (Fig. 1). A retrograde ureteropyelogram was obtained (Fig. 2). The LithoVue was then inserted using 12/14F ureteral access sheath (Olympus, Spencer, IN). Endoscopy revealed two stones overlying the left lower pole calix. The two stones were then observed in the lower pole calix that were covered in a “mossy fibrinous-like material consistent with struvite stones.” A 200 μm Ho:YAG fiber at the time was not available; therefore, a 365 μm fiber was inserted into the ureteroscope and advanced toward the stones (Fig. 3). The laser was used for more than 4000 pulses at 15 W, producing mucoid debris and fragments. A 1.9F ZeroTip basket was used to extract the stone remains, and the patient was rendered stone free. Treatment success was confirmed by abdominal film obtained 1 week after stent removal.

## Discussion

Causes of ureteroscope failure and damage result from a number of cases performed, time spent in use, and the overall time spent in the lower pole.^2^ Renal stones located in the lower pole of the kidney can be extremely challenging as extreme deflections of greater than 160° are difficult to maintain and are often further compromised when using stone treatment tools, such as laser fibers and baskets.^7^ Lower pole stones place ureteroscopes at a greater risk of damage because of increased deflection.^2^ As deflection increases, damage occurs through thermal breakdown resulting from the leakage of laser energy into the cladding and external sheath.^8^ Therefore, the smallest diameter fibers are required for maximized deflection and laser energy flow.^8^ Other digital ureteroscopes, as demonstrated by Ketul et al., are large, >8F tip, and often require an extra step of using a basket to reposition a lower pole stone in the upper pole before removal.^3^ Both the DUR-D (ACMI, Marlborough, MA) and URF-V (Olympus Corporation of the Americas, Center Valley, PA) digital ureteroscopes were ineffective in reaching lower pole kidney stones, in the study described by Ketul et al. However, the smaller URF-P5 Olympus flexible fiber-optic ureteroscope was effective in each case. This, therefore, requires the additional costs of purchasing and maintaining a smaller diameter fiber-optic ureteroscope(s) for the cases where the reusable digital ureteroscope is insufficient.^3^ In comparison with these larger digital ureteroscopes, the 7.7F tip diameter of the LithoVue is relatively small allowing for effective lithotripsy of hard-to-reach lower pole calculi, eliminating the potential need and acquired costs of an alternative fiber-optic ureteroscope, while maintaining high image resolution.^6^

## Conclusion

The LithoVue system single-use digital flexible ureteroscope provides an economical advantage over both reusable digital and fiber-optic ureteroscopes. The LithoVue system uses the enhanced image resolution of the digital CMOS imager, similar to other reusable digital ureteroscopes, while maintaining the small ureteroscope size of a flexible fiber-optic ureteroscope, allowing for consistent and effective lower pole access. Deflection characteristics are maintained even when thicker laser fibers are passed through the working channel.

## Abbreviation Used

UTI: urinary tract infections
